# Responsible leadership and sustainability performance in health systems: evidence from health and sanitation projects

**DOI:** 10.3389/frhs.2026.1866029

**Published:** 2026-07-23

**Authors:** Mahadih Kyambade, Rogers Mwesigwa, Kassim Alinda, Sulait Tumwine, Freddie Lwanga

**Affiliations:** 1 Department of Leadership and Governance, Makerere University Business School, Kampala, Uganda; 2 Department of Business Administration, Makerere University Business School, Kampala, Uganda; 3 Department of Accounting, Makerere University Business School, Kampala, Uganda; 4 Department of Human Resource Management, Makerere University Business School, Kampala, Uganda

**Keywords:** accountability and transparency, ethical decision-making, health and sanitation projects, long-term orientation, responsible leadership, sustainability performance

## Abstract

**Purpose:**

This study examines the influence of responsible leadership on the sustainability performance of health and sanitation projects in Uganda. Specifically, the study investigates how ethical decision-making, accountability and transparency, and long-term orientation predict triple bottom line sustainability outcomes (economic, social, and environmental performance).

**Methodology:**

A cross-sectional quantitative design was employed using data collected from 520 stakeholders across 86 health and sanitation projects in Kampala, Wakiso, and Mukono districts. Data were analyzed using Partial Least Squares Structural Equation Modeling (PLS-SEM) with SmartPLS 4.

**Findings:**

The findings reveal that responsible leadership significantly and positively influences sustainability performance. Furthermore, ethical decision-making, accountability and transparency, and long-term orientation each independently and positively predict economic, social, and environmental sustainability outcomes. The results underscore the central role of relational leadership behaviors in enhancing project sustainability beyond structural governance mechanisms.

**Practical implications:**

Practically, the study suggests that policymakers, donors, and implementing agencies should institutionalize responsible leadership competencies within project management systems through training, accountability frameworks, and long-term sustainability planning.

**Originality:**

This study is original in that it extends responsible and relational leadership theory into the health and sanitation project context in Sub-Saharan Africa, a sector largely overlooked in prior sustainability research. By empirically linking leadership behaviors to triple bottom line outcomes in public health projects, the study provides context-specific evidence for advancing sustainable development in line with global sustainability goals.

## Introduction

Sustainability performance has become a central concern in contemporary development discourse, particularly in sectors directly linked to human well-being such as health and sanitation. Sustainable health and sanitation systems are essential for improving population health outcomes, strengthening institutional capacity, and safeguarding environmental resources for future generations. Globally, the urgency of sustainability in these sectors is reinforced by the United Nations Sustainable Development Goals (SDGs), especially SDG 3, which seeks to ensure healthy lives and promote well-being for all, and SDG 6, which aims to ensure availability and sustainable management of water and sanitation for all. Achieving these goals requires projects that deliver not only short-term outputs but also long-term economic viability, social inclusion, and environmental protection. As highlighted in recent reviews, sustainability performance must be assessed through a triple bottom line lens encompassing economic, social, and environmental dimensions (1–3). In this regard, strengthening sustainability performance in health and sanitation projects is not merely a managerial priority but a global development imperative.

The need for sustainable performance in health and sanitation projects is particularly pressing in low- and middle-income countries where access to safe water, sanitation, and quality healthcare remains uneven. According to the World Health Organization and UNICEF Joint Monitoring Programme, approximately 3.5 billion people globally lack safely managed sanitation services, while more than 2 billion people do not have access to safely managed drinking water services (4, 6). In Sub-Saharan Africa, inadequate sanitation and weak health infrastructure continue to contribute significantly to disease burdens, environmental degradation, and social inequalities. Uganda faces similar challenges, with rapid urbanization, population growth, and infrastructure deficits placing increasing pressure on health and sanitation systems. Recent national statistics indicate that access to safely managed sanitation services remains limited, particularly in peri-urban and rural communities, while many water and sanitation interventions struggle to maintain functionality beyond donor support periods (5–7). These challenges underscore the importance of improving the sustainability performance of health and sanitation projects to ensure long-term service delivery and developmental impact.

Across the globe, leadership has increasingly been recognized as a critical driver of sustainable performance. The concept of responsible leadership grounded in ethics, accountability, and long-term value creation, has gained prominence in both theory and practice (8–10). Empirical evidence shows that responsible leadership enhances financial and environmental performance (11), promotes sustainable organizational outcomes (12), and strengthens pro-environmental behaviors and green innovation (13). In project-based settings, responsible leadership has been linked to improved stakeholder collective performance (14) and project social responsibility outcomes (15). These findings suggest that leaders who prioritize ethical decision-making, transparency, accountability, and long-term orientation are more likely to influence sustainable performance positively. However, much of this evidence originates from corporate, manufacturing, construction, and hospitality contexts, with limited attention to health and sanitation projects, particularly in developing economies.

In the African context, sustainability challenges remain acute due to institutional weaknesses, resource constraints, and governance gaps. Studies in Uganda have examined sustainability performance in sectors such as power companies (16, 17), manufacturing firms (18), and SMEs (19), emphasizing factors such as stakeholder orientation, management control systems, and human capital. While these studies contribute valuable insights into sustainable performance in corporate and industrial sectors, they largely overlook project-based interventions in health and sanitation. Recent evidence indicates that Uganda's health and sanitation projects face persistent sustainability challenges, including weak institutional coordination, limited accountability mechanisms, and inadequate long-term planning (1, 20). Although stakeholder engagement and resilience have been explored within this sector (21), the explicit role of responsible leadership as a predictor of sustainability performance remains underexplored. This gap is particularly concerning given the centrality of leadership in shaping ethical standards, transparency, and strategic orientation in public and donor-funded projects.

Despite substantial investments in health and sanitation infrastructure across Africa, sustainability outcomes remain inconsistent due to governance challenges, institutional weaknesses, resource constraints, and limited accountability mechanisms. Studies conducted across developing economies demonstrate that project sustainability is frequently undermined by inadequate leadership, poor stakeholder coordination, weak monitoring systems, and insufficient long-term planning (22–24). In Uganda, health and sanitation initiatives continue to face challenges related to service continuity, maintenance of infrastructure, financial sustainability, and institutional coordination, particularly after the withdrawal of external funding (4, 5). While existing Ugandan studies have examined sustainability performance within manufacturing firms, SMEs, energy utilities, and other organizational contexts (3, 16–19), relatively little attention has been given to project-based health and sanitation interventions where sustainability outcomes directly affect public welfare and environmental health. Consequently, the mechanisms through which leadership practices influence sustainability performance within these projects remain insufficiently understood.

Although responsible leadership has increasingly been associated with sustainability outcomes, most empirical evidence originates from corporate, manufacturing, hospitality, and construction settings (25–28). Existing studies primarily examine sustainability performance at the organizational or firm level and pay limited attention to project-based environments characterized by multiple stakeholders, donor involvement, public accountability, and complex social objectives. Furthermore, evidence from Sub-Saharan Africa remains relatively scarce, despite the region facing some of the most significant sustainability challenges in health and sanitation service delivery. As a result, there is limited empirical understanding of how responsible leadership dimensions such as ethical decision-making, accountability and transparency, and long-term orientation influence economic, social, and environmental sustainability performance within health and sanitation projects. Addressing this gap, the present study investigates the relationship between responsible leadership and sustainability performance in Ugandan health and sanitation projects, thereby extending responsible leadership scholarship into an underexplored public-sector project context while contributing to the achievement of SDGs 3 and 6.

## Literature review

### Theoretical review

Relational Leadership Theory (RLT), as advanced by Uhl-Bien (29), conceptualizes leadership as a social influence process embedded in relationships, interactions, and networks rather than merely in formal authority or individual traits. Leadership effectiveness, therefore, emerges from trust, shared meaning-making, mutual accountability, and collaborative engagement among stakeholders. This relational perspective aligns closely with sustainability performance, which depends on coordinated actions among diverse actors to achieve economic, social, and environmental outcomes.

This view complements responsible leadership scholarship, which positions leaders as ethical relationship-builders within a stakeholder society (30, 31). Responsible leadership emphasizes moral accountability, stakeholder engagement, and long-term value creation (10, 32, 33). By fostering dialogue, transparency, and shared responsibility, responsible leaders create the relational conditions necessary for sustainable performance. In project-based environments, relational dynamics are especially critical, as sustainability outcomes depend on multi-stakeholder coordination (14).

In Uganda's health and sanitation projects where sustainability challenges include weak coordination and limited long-term planning (1, 20), RLT provides a useful lens for explaining how ethical decision-making, accountability and transparency, and long-term orientation function as relational mechanisms that strengthen stakeholder trust and collective commitment. Thus, RLT underpins this study by explaining how responsible leadership behaviors, enacted through high-quality relationships, predict sustainability performance across triple bottom line dimensions.

### Responsible leadership practices and sustainability performance

Responsible leadership has emerged as a relational and stakeholder-oriented leadership paradigm that integrates ethics, accountability, and long-term value creation (8–10). It emphasizes balancing economic objectives with social and environmental responsibilities, consistent with the triple bottom line framework (2). Empirical studies increasingly demonstrate that responsible leadership enhances sustainable performance outcomes. For instance, Huo et al. (25) found that responsible leadership positively influences sustainable performance through complex mediation mechanisms, while Nakra and Kashyap (12) reported that responsible leadership strengthens organizational sustainability performance via sustainable HRM practices. Similarly, Ur Rehman et al. (11) linked responsible leadership to improved financial and environmental performance, and Liu et al. (13) showed that responsible leadership fosters sustainable business performance through green innovation and pro-environmental behavior.

Project-based studies also confirm this relationship. Lin et al. (14) demonstrated that responsible leadership enhances stakeholder collective performance in construction projects, while Yu et al. (15) and Maqsoom et al. (27) linked responsible leadership to social responsibility performance and pro-environmental behaviors in project settings. However, most of these studies are situated in manufacturing, construction, hospitality, or corporate environments. In Uganda, sustainability performance research has focused on corporate disclosures (3), power companies (16, 17), manufacturing firms (18), and SMEs (19), largely overlooking leadership as a direct predictor and neglecting health and sanitation projects. Although recent studies have documented sustainability challenges in Uganda's health and sanitation projects (1, 20), empirical evidence linking responsible leadership to sustainability performance in this sector remains scarce. This gap necessitates examining responsible leadership as a direct predictor of triple bottom line sustainability performance within health and sanitation projects.

H1: Responsible leadership practices, represented by ethical decision-making, accountability and transparency, and long-term orientation combined, positively and significantly influences sustainability performance of health and sanitation projects.

### Ethical decision-making and sustainability performance

Ethical decision-making constitutes a core dimension of responsible leadership, reflecting leaders' commitment to fairness, integrity, and moral accountability in stakeholder interactions (34, 35). Ethical leaders foster ethical climates that promote responsible behaviors and long-term sustainability outcomes (26). Empirical research indicates that ethical leadership behaviors enhance environmental and social performance by encouraging employees' pro-environmental conduct and adherence to sustainability standards (36, 37). In the sustainability domain, Abraham (2) argues that ethical orientation is central to achieving triple bottom line outcomes, as it guides responsible resource allocation and stakeholder engagement.

Despite this evidence, most studies examine ethical leadership within corporate or industrial settings, focusing on firm-level performance rather than project-based public health interventions. Moreover, while sustainability drivers in Uganda have been linked to governance mechanisms and human capital (16, 17), limited attention has been given to how ethical decision-making by project leaders shapes sustainability performance in health and sanitation initiatives. Given the complex ethical challenges in public health projects including equitable access, transparency in resource use, and environmental protection, there is a need to empirically test how ethical decision-making predicts economic, social, and environmental sustainability outcomes in this sector.

H2: Ethical decision-making positively and significantly influences sustainability performance of health and sanitation projects.

### Accountability, transparency, and sustainability performance

Accountability and transparency are central to responsible leadership, reinforcing trust, legitimacy, and stakeholder confidence (9, 38). Leaders who demonstrate transparency in decision-making and reporting foster stronger governance systems and improved sustainability outcomes. Empirical evidence suggests that transparent and accountable leadership enhances environmental and financial performance by reducing opportunistic behavior and strengthening stakeholder alignment (11, 25). Muff et al. (39) further identify accountability and stakeholder engagement as key competencies of responsible leaders driving sustainable organizational performance.

In Uganda, sustainability performance research has emphasized regulatory governance and management control systems (16, 17) and sustainability disclosures (3), highlighting structural accountability mechanisms. However, these studies focus on institutional systems rather than leader-driven accountability and transparency behaviors. In health and sanitation projects where misallocation of funds, weak reporting, and limited oversight can undermine sustainability, leadership accountability may play a decisive role in ensuring long-term viability (20). Yet, empirical studies explicitly linking accountability and transparency as leadership behaviors to sustainability performance in this sector remain limited.

H3: Accountability and transparency positively and significantly influence sustainability performance of health and sanitation projects.

### Long-term orientation and sustainability performance

Long-term orientation reflects a leader's commitment to future-focused planning, intergenerational equity, and sustained value creation (40). Responsible leaders adopt systems thinking and prioritize long-term stakeholder welfare over short-term gains (8, 41). Empirical studies show that leadership approaches emphasizing long-term sustainability enhance environmental innovation and sustained financial performance (13, 42). In project contexts, leaders who adopt a long-term orientation are more likely to integrate sustainability considerations into strategic planning and stakeholder management (14, 43).

However, much of the evidence originates from corporate, manufacturing, or hospital management contexts. Limited research has examined long-term orientation in donor-funded or government-supported health and sanitation projects in developing countries. In Uganda, sustainability challenges in health and sanitation projects are often linked to short-term funding cycles and inadequate long-term planning (1, 20). While structural drivers of sustainability have been identified (1), the role of leaders' long-term orientation in shaping sustainability performance remains underexplored. Therefore, examining long-term orientation as a predictor of triple bottom line sustainability performance in health and sanitation projects contributes to filling this contextual and sectoral gap.

H4: Long-term orientation positively and significantly influences sustainability performance of health and sanitation projects.

## Methodology

### Research design

This study employed a cross-sectional quantitative research design to examine the predictive relationship between responsible leadership and sustainability performance in health and sanitation projects in Central Uganda. A cross-sectional design collects data from a defined sample at a single point in time to examine relationships among variables and test theoretical propositions (44). This design was appropriate because the study sought to test hypothesized associations without manipulating variables and to generate generalizable findings across multiple projects. A quantitative approach enabled statistical modeling and hypothesis testing using Partial Least Squares Structural Equation Modeling (PLS-SEM), consistent with prediction-oriented research designs (45).

### Study area and population

The study was conducted in Central Uganda, specifically in the districts of Kampala, Wakiso, and Mukono. These districts were selected due to their high concentration of public, donor-funded, and NGO-supported health and sanitation projects and their strategic importance in Uganda's service delivery landscape. The study population consisted of 110 health and sanitation projects, including both ongoing and recently completed projects within the three districts. The research adopted a two-level population structure. The unit of analysis was the health and sanitation project because the study sought to examine sustainability performance at the project level, including how leadership practices influence the long-term economic, social, and environmental outcomes of project implementation. Health and sanitation projects were therefore considered the primary entities through which sustainability performance could be assessed and compared. The unit of inquiry comprised stakeholders directly involved in, overseeing, implementing, or benefiting from these projects, as they possessed relevant knowledge and experience regarding project leadership, management practices, and sustainability outcomes. These stakeholders included project managers, engineers, public health officers, monitoring and evaluation staff, finance/procurement officers, district technical personnel, and beneficiary representatives. This dual-level structure ensured that sustainability performance was assessed at the project level while data were collected from knowledgeable project actors.

### Sample size determination and sampling procedure

The sample size for projects was determined using Yamane's formula for finite populations (46). The formula is given as: *n* = N/ (1 + N(e)^2^, where *n* is the sample size, *N* is the population size, and *e* is the level of precision (0.05). Therefore: *n* = 110/(1 + 110(5%)^2^ = 86 projects were included in the study. Consequently, 86 projects were selected from the 110 projects using stratified sampling based on the districts of Kampala, Wakiso, and Mukono to ensure proportional representation across the study area. Specifically, Kampala contributed 31 projects; Wakiso 35 projects; and Mukono contributed 20 projects, giving a total of 86 projects from the overall population. In addition, an *a priori* power analysis using G-Power 3.1, assuming a medium effect size (f² = 0.15), significance level of *α* = 0.05, and statistical power of 0.80, indicated a minimum requirement of approximately 92 respondents from these projects.

From the 86 selected projects, a total of 600 questionnaires were distributed. Out of these, 520 valid responses were received and used for analysis, representing a response rate of 86.7%. Therefore, the sample of 520 respondents substantially exceeds the G-Power threshold, demonstrating the adequacy, robustness, and statistical effectiveness of the study in ensuring reliable, precise, and generalizable findings. Within each project, stakeholders were purposively selected based on their involvement in leadership processes, sustainability planning, financial oversight, environmental management, or community engagement (47). The final sample size of 520 respondents also exceeds the minimum requirements for PLS-SEM and enhances model stability, predictive accuracy, and generalizability (45).

Although health and sanitation projects constituted the unit of analysis, data were collected from individual stakeholders serving in managerial, technical, governance, and beneficiary-representative roles. Accordingly, the study analyzed stakeholder-based assessments of project characteristics and outcomes at the individual level rather than aggregating responses to the project level.

### Questionnaire development

Data were collected using a self-administered structured questionnaire composed of closed-ended items measured on a five-point Likert scale ranging from 1 (strongly disagree) to 5 (strongly agree). The five-point scale was chosen because it balances measurement sensitivity and simplicity, making it suitable for respondents with diverse professional and educational backgrounds (48). It provides sufficient variability for robust statistical analysis while minimizing respondent fatigue and cognitive burden (49). Moreover, five-point scales are widely accepted in PLS-SEM research and generate adequate variance for reliable structural modeling (45, 57). Several procedural remedies were implemented to minimize common method bias. Respondents were assured of anonymity and confidentiality, participation was voluntary, and questionnaire items were carefully structured and organized to reduce evaluation apprehension and response consistency tendencies. Clear instructions were provided, and respondents were encouraged to answer honestly based on their project experiences.

### Measurement of variables

Responsible Leadership (RL) was operationalized as a multidimensional reflective construct comprising such as Ethical decision-making, Accountability and transparency, and Long-term orientation. This operationalization was grounded in responsible leadership scholarship (2, 8, 10, 34). Measurement items were adapted from established scales reported in prior responsible leadership and sustainability literature. Minor wording modifications were made to align the items with the health and sanitation project context in Uganda while preserving their original conceptual meaning. Ethical Decision-Making was measured using four items (e.g., “Project leaders make decisions based on ethical principles”), Accountability and Transparency using four items (e.g., “Project leaders communicate project information openly to stakeholders”), and Long-Term Orientation using four items (e.g., “Project leaders prioritize long-term project sustainability over short-term gains”). Whereas Sustainability Performance (SP) was measured using the triple bottom line dimensions such as economic sustainability, social sustainability, and environmental sustainability. This measurement approach aligns with prior sustainability performance literature (2, 13, 50). Sample items were, Economic Sustainability was measured using three items (e.g., “The project utilizes financial resources efficiently to ensure long-term viability”), Environmental Sustainability using two items (e.g., “The project minimizes negative environmental impacts during implementation”), and Social Sustainability using three items (e.g., “The project contributes positively to community well-being and stakeholder welfare”).

### Validity and reliability assessment

Content validity was established through consultation with an expert panel comprising academics, sustainability scholars, and public health practitioners. Experts rated the clarity and relevance of each item using a five-point scale. The computed Content Validity Index (CVI) values exceeded the recommended threshold of 0.70, indicating satisfactory content validity (49). Internal consistency reliability was assessed using Cronbach's alpha and Composite Reliability (CR), with acceptable thresholds above 0.70 (45, 51). Convergent validity was evaluated through Average Variance Extracted (AVE), with values above 0.50 indicating adequate convergence (45, 52). Discriminant validity was assessed using the Fornell–Larcker criterion and the Heterotrait–Monotrait (HTMT) ratio to ensure construct distinctiveness. In addition to content validity assessment, an exploratory factor analysis (EFA) was conducted as a preliminary step to examine the underlying factor structure of the adapted measurement scales within the Ugandan health and sanitation project context. Given that the scales were adapted from prior studies and applied in a different contextual setting, EFA was used to confirm dimensionality before proceeding to confirmatory assessment through PLS-SEM.

### Data analysis

Hypotheses were tested using Partial Least Squares Structural Equation Modeling (PLS-SEM) with SmartPLS 4. PLS-SEM was chosen because it is prediction-oriented, robust to non-normal data, and suitable for complex hierarchical models (45, 53, 57). Common method bias was assessed using the full collinearity variance inflation factor (VIF) approach. According to (54), VIF values below 3.3 indicate that common method bias is unlikely to be a serious concern. As presented in Table 1, all VIF values were below the recommended threshold, suggesting that common method bias did not materially affect the study results. The analytical procedure followed a two-step approach. First, the measurement model was evaluated by assessing indicator reliability, internal consistency reliability, convergent validity, and discriminant validity (45). Second, the structural model was assessed by examining collinearity (VIF < 3.3), path coefficients (*β*), statistical significance using bootstrapping with 5,000 resamples, coefficient of determination (R²), effect sizes (f²), and predictive relevance (Q²). Responsible leadership was conceptualized as a multidimensional leadership construct comprising ethical decision-making, accountability and transparency, and long-term orientation. For the purposes of structural model estimation, the three dimensions were modeled as separate first-order constructs to examine their individual contributions to sustainability performance. This approach enabled the identification of the relative influence of each responsible leadership dimension while preserving the multidimensional conceptualization of responsible leadership. The predictive accuracy of the model was further examined using the PLSpredict procedure.

**Table 1:** Reliability and validity of constructs.

Construct	Cronbach's Alpha	Composite Reliability	AVE	VIF
Ethical Decision Making	0.842	0.887	0.612	1.352
Accountability & Transparency	0.836	0.878	0.603	1.414
Long-term Orientation	0.865	0.903	0.621	1.528
Responsible Leadership	0.889	0.912	0.677	1.621
Economic Sustainability	0.821	0.867	0.571	1.403
Environmental Sustainability	0.807	0.851	0.546	1.466
Social Sustainability	0.829	0.873	0.559	1.381
Sustainability Performance	0.914	0.928	0.685	1.554

Source: Author's own work.

All VIF values are below 3.3, indicating the absence of multicollinearity.

## Results

### Respondent characteristics

The study analysed responses from 520 participants involved in the implementation and management of health and sanitation projects (see Table 2). The respondents consisted mainly of project managers, engineers, monitoring and evaluation officers, environmental officers, procurement specialists, and field coordinators working in health and sanitation infrastructure initiatives across Uganda.

**Table 2:** Respondent characteristics (*n* = 520).

Variable	Category	Frequency	Percentage (%)
Gender	Male	312	60.0
	Female	208	40.0
Age	Below 35	142	27.3
	36–45	248	47.7
	46–55	101	19.4
	Above 55	29	5.6
Education	Bachelor's degree	304	58.5
	Master's degree	176	33.8
	PhD	40	7.7

Source: Author's own work.

The study analyzed responses from 520 participants involved in health and sanitation projects. The majority of respondents were male (60.0%), while females constituted 40.0%. Most respondents were between 36 and 45 years of age (47.7%), suggesting that the projects are largely managed by mature professionals with substantial experience. Regarding education level, the majority held bachelor's degrees (58.5%), followed by master's degrees (33.8%), indicating that most respondents possess adequate academic qualifications to understand sustainability and leadership practices within project environments.

### Measurement model assessment

Following the recommendations of (45), the measurement model was evaluated using indicator reliability, internal consistency reliability, convergent validity, and discriminant validity.

### Indicator reliability

**Figure 1:**
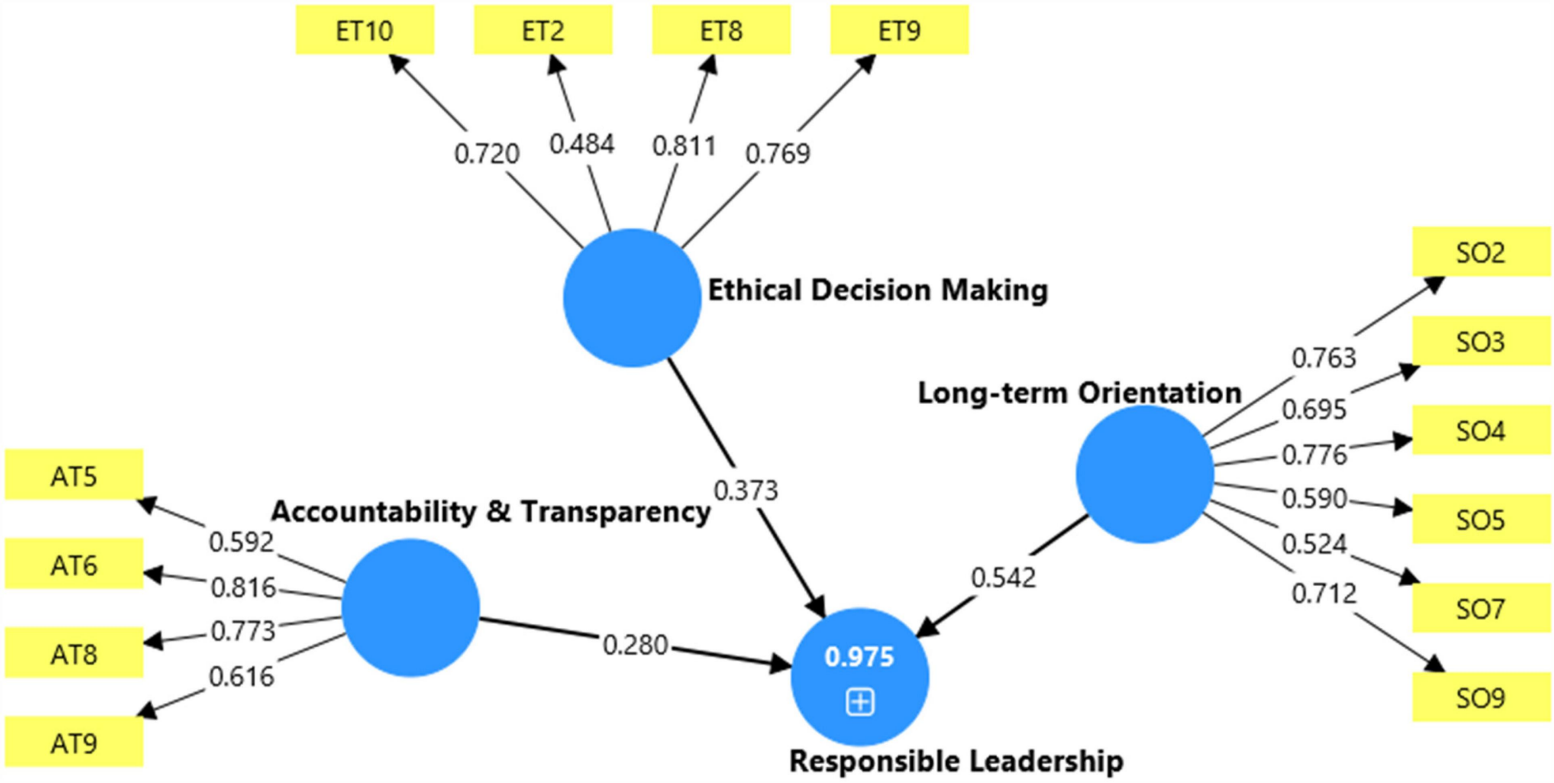
Responsible Leadership Measurement Model. Source: Author's own work.

Indicator reliability was assessed using outer loadings obtained from the PLS measurement model. The majority of indicators exhibited loadings above the recommended threshold of 0.70, while a few items slightly below this threshold were retained because they contributed meaningfully to construct validity. The measurement model for responsible leadership is presented in Figure 1, which illustrates the relationships between the dimensions of responsible leadership including ethical decision making, accountability and transparency, and long-term orientation.

Figure 1 presents the measurement model for responsible leadership and its underlying dimensions, namely ethical decision making, accountability and transparency, and long-term orientation. The outer loadings displayed in the model indicate that the observed indicators are strongly associated with their respective latent constructs. Most indicator loadings exceed the recommended threshold of 0.60, confirming that the measurement items reliably capture the constructs they represent. Among the three dimensions, long-term orientation shows relatively strong indicator loadings, suggesting that it is a particularly stable component of responsible leadership within health and sanitation project management.

**Figure 2:**
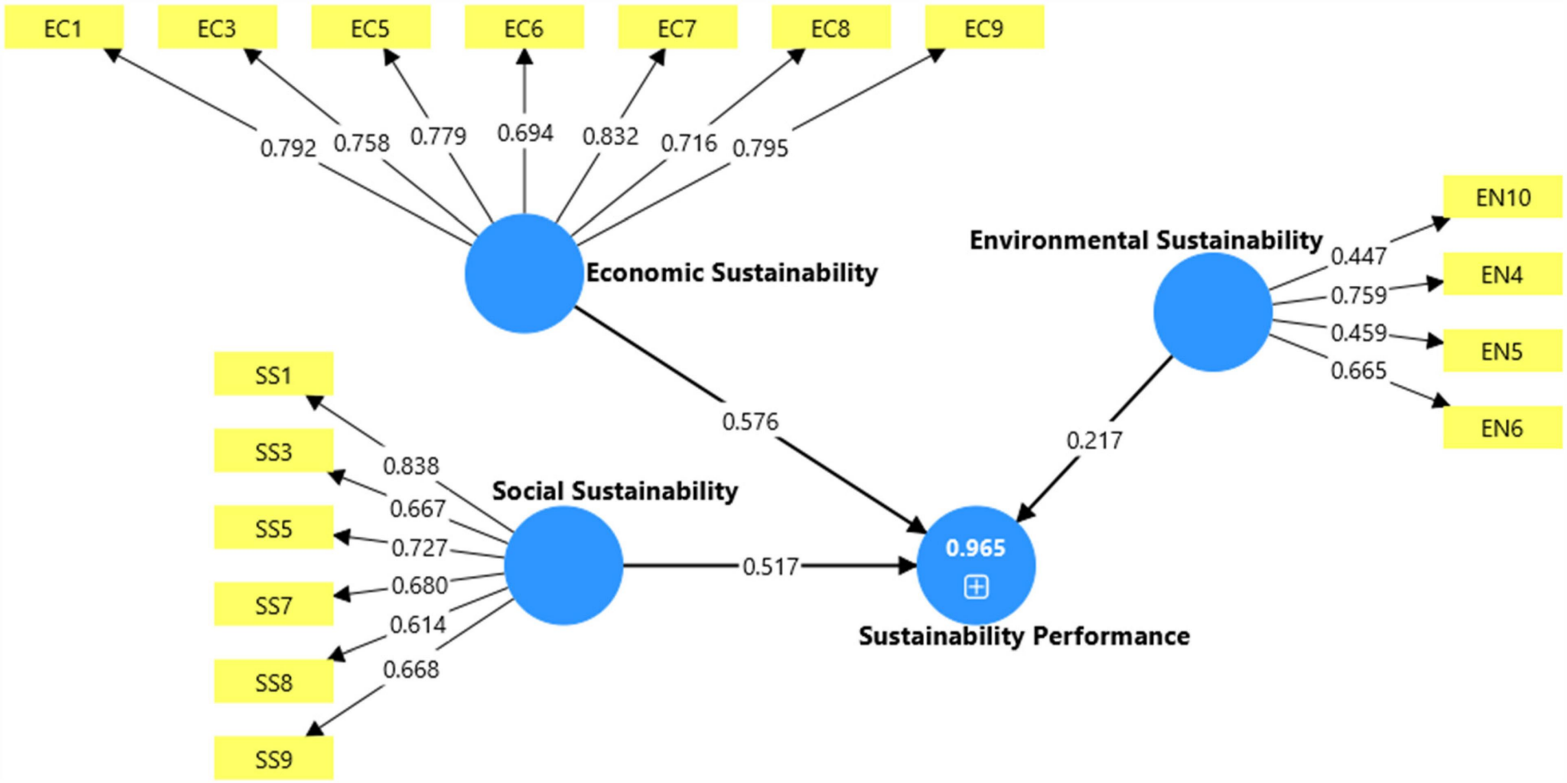
Sustainability Performance Measurement Model. Source: Author's own work.

The measurement model for sustainability performance is presented in Figure 2, which illustrates the relationships between the dimensions of sustainability performance including economic, social and environmental sustainability performance.

Figure 2 illustrates the measurement model for sustainability performance, which is operationalized through economic, environmental, and social sustainability dimensions. The outer loadings of the indicators demonstrate that the measurement items adequately represent their respective constructs. Most indicators exceed the recommended loading threshold, indicating acceptable reliability and convergent validity. The results suggest that sustainability performance in health and sanitation projects is effectively captured through these three interrelated dimensions.

### Reliability and convergent validity

Reliability and convergent validity were assessed using Cronbach's alpha, composite reliability (CR), and average variance extracted (AVE). The results presented in Table 1 indicate that all constructs meet these criteria, confirming satisfactory reliability and convergent validity.

Variance inflation factor (VIF) values were examined to assess potential collinearity issues among predictor constructs. According to Kock (54), VIF values should be below 3.3 to confirm the absence of multicollinearity. As shown in Table 1, all VIF values are well below the recommended threshold. This indicates that collinearity does not pose a concern in the structural model.

### Discriminant validity

Discriminant validity was examined using the Fornell–Larcker criterion, which requires that the square root of AVE for each construct should be greater than its correlations with other constructs (55). The results presented in Table 3 demonstrate that this condition is satisfied, confirming that each construct is empirically distinct.

**Table 3:** Fornell–Larcker criterion.

Construct	RL	ET	AT	SO	SP
Responsible Leadership (RL)	**0**.**823**				
Ethical Decision Making (ET)	0.621	**0**.**782**			
Accountability & Transparency (AT)	0.593	0.512	**0**.**776**		
Long-term Orientation (SO)	0.667	0.538	0.521	**0**.**788**	
Sustainability Performance (SP)	0.698	0.546	0.501	0.612	**0.828**

Source: Author's own work.

Diagonal values represent the square root of AVE.

Note: Bold diagonal values represent the square root of the Average Variance Extracted (AVE) for each construct. Discriminant validity is supported when these values exceed the corresponding inter-construct correlations.

The heterotrait–monotrait ratio (HTMT) was used to further assess discriminant validity. According to Henseler et al. (45) and Hair et al. (58), HTMT values should be below 0.90 to confirm that constructs are empirically distinct. As shown in Table 4, all HTMT values range between 0.541 and 0.756, which are well below the recommended threshold. This indicates that each construct captures a unique conceptual domain and that discriminant validity is satisfactorily established for the measurement model.

**Table 4:** HTMT discriminant validity results.

Constructs	EDM	AT	SO	RL	EC	EN	SS	SP
Ethical Decision Making (ET)	—							
Accountability & Transparency (AT)	0.684	—						
Long-term Orientation (SO)	0.712	0.676	—					
Responsible Leadership (RL)	0.742	0.721	0.764	—				
Economic Sustainability (EC)	0.601	0.584	0.633	0.657	—			
Environmental Sustainability (EN)	0.567	0.541	0.612	0.628	0.673	—		
Social Sustainability (SS)	0.589	0.557	0.621	0.644	0.681	0.659	—	
Sustainability Performance (SP)	0.623	0.598	0.681	0.714	0.756	0.701	0.743	—

Source: Author's own work.

Model fit indices were assessed to determine how well the structural model reproduces the observed covariance matrix as shown in Table 5 above. The SRMR value of 0.061 is below the recommended threshold of 0.08, indicating good model fit. Similarly, the NFI value of 0.912 exceeds the recommended minimum of 0.90, suggesting that the proposed model adequately fits the data. In addition, the RMS_theta value of 0.082 is below the recommended threshold of 0.12, indicating that the outer model residuals are minimal. Collectively, these indices confirm that the structural model demonstrates acceptable global model fit and predictive accuracy. Although the study employed established scales drawn from prior responsible leadership and sustainability performance literature, EFA was performed as an initial diagnostic procedure to assess the suitability of the adapted items for the study context. The subsequent PLS-SEM measurement model assessment provided a more rigorous evaluation of reliability and validity.

**Table 5:** Model fit indices.

Model Fit Indicator	Value	Recommended Threshold	Interpretation
SRMR	0.061	< 0.08	Good fit
NFI (Normed Fit Index)	0.912	> 0.90	Acceptable fit
RMS_theta	0.082	< 0.12	Good fit

Source: Author's own work.

### Sampling adequacy and factor analysis

Prior to conducting factor analysis, the Kaiser–Meyer–Olkin (KMO) test and Bartlett's test of sphericity were conducted to assess the suitability of the data for factor analysis. The results presented in Table 6 shows that the KMO values exceeded the recommended threshold of 0.70, and Bartlett's test was statistically significant (*p* < 0.001), indicating that the dataset was appropriate for factor analysis.

**Table 6:** KMO and Bartlett's test.

Construct	KMO	Chi-Square	df	Sig
Responsible Leadership	0.812	1,356.44	210	0.000
Sustainability Performance	0.794	921.67	105	0.000

Source: Author's own work.

### Exploratory factor analysis

Exploratory factor analysis was conducted using principal component analysis with varimax rotation. The results indicate that the extracted factors explain a substantial proportion of the total variance, exceeding the minimum threshold of 60% recommended in social science research.

The results in Table 7 indicate that the three factors representing responsible leadership such as ethical decision making, accountability and transparency, and long-term orientation collectively explain 68.4% of the total variance, which exceeds the 60% threshold commonly recommended for social science research (45).

**Table 7:** Exploratory factor analysis for responsible leadership.

Item	Ethical Decision Making	Accountability	Long-term Orientation
ET10	0.720		
ET8	0.811		
ET9	0.769		
AT6		0.816	
AT8		0.773	
AT9		0.616	
SO2			0.763
SO3			0.695
SO4			0.776
SO9			0.712
Eigenvalues	3.241	2.108	1.491
Variance %	32.41	21.08	14.91
Cumulative %	32.41	53.49	68.40

Source: Author's own work.

ET, ethical decision-making items; AT, accountability and transparency items; SO, long-term orientation items.

The extracted factors in Table 8 represent sustainability performance that is economic sustainability, environmental sustainability, and social sustainability explain 71.2% of the total variance, indicating strong construct validity and supporting the multidimensional structure of sustainability performance.

**Table 8:** Exploratory factor analysis for sustainability performance.

Item	Economic	Environmental	Social
EC1	0.792		
EC3	0.758		
EC5	0.779		
EN4		0.759	
EN6		0.665	
SS1			0.838
SS3			0.667
SS5			0.727
Eigenvalues	3.412	1.978	1.306
Variance %	34.12	19.78	17.30
Cumulative %	34.12	53.90	71.20

Source: Author's own work.

EC, economic sustainability items; EN, environmental sustainability items; SS, social sustainability items.

## Structural model results

### Economic sustainability model

**Figure 3:**
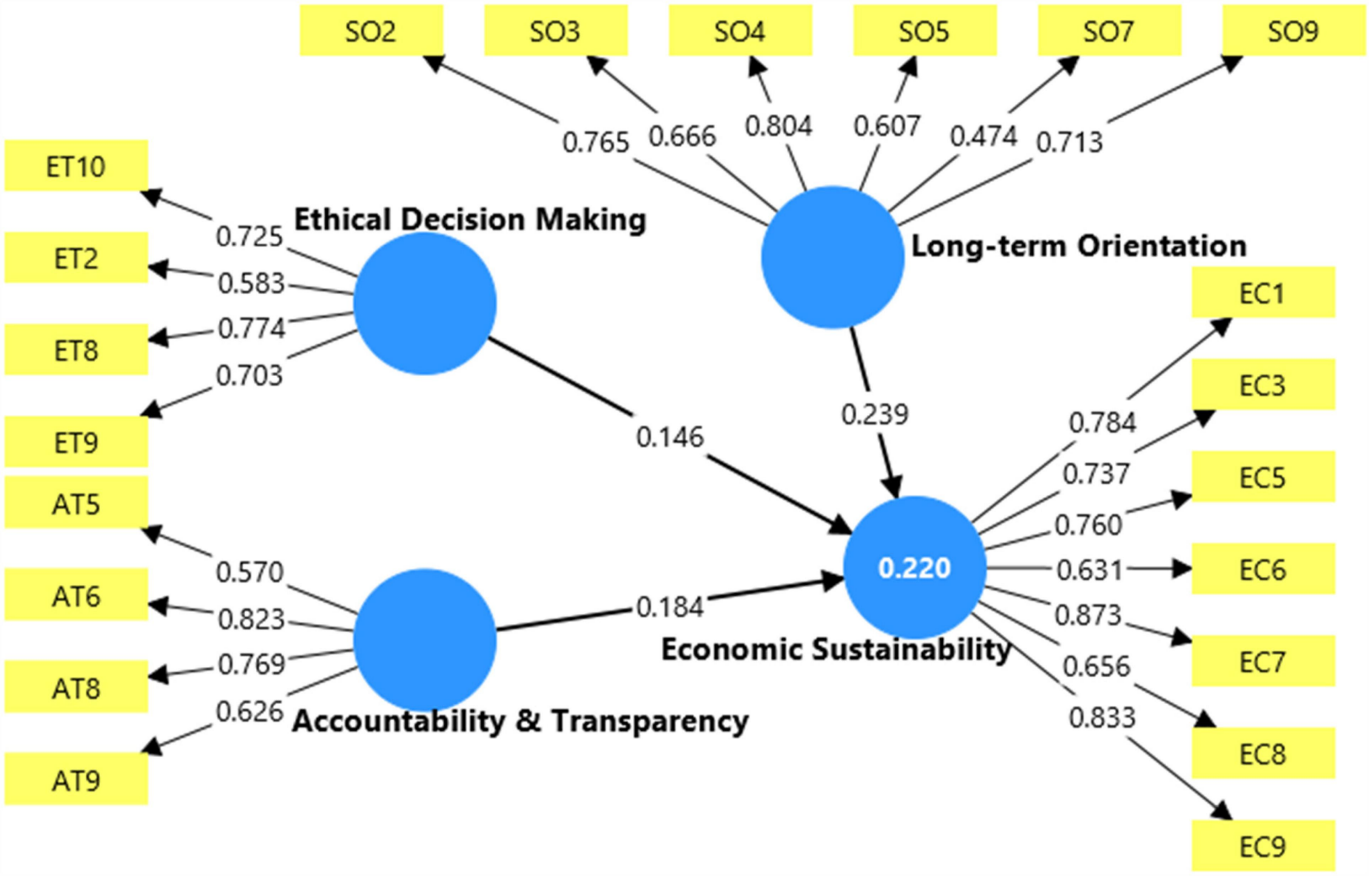
Economic Sustainability model. Source: Author's own work.

The predictive relationships between responsible leadership and economic sustainability are illustrated in Figure 3.

Figure 3 shows the predictive relationships between responsible leadership dimensions and economic sustainability. The results indicate that responsible leadership explains 22.0% of the variance in economic sustainability (R² = 0.220). Among the leadership dimensions, long-term orientation emerges as the strongest predictor, indicating that leaders who emphasize strategic planning and future-oriented decision making contribute more significantly to improving the economic viability of health and sanitation projects. Ethical decision making and accountability also contribute positively, although their predictive effects are comparatively smaller.

### Environmental sustainability model

**Figure 4:**
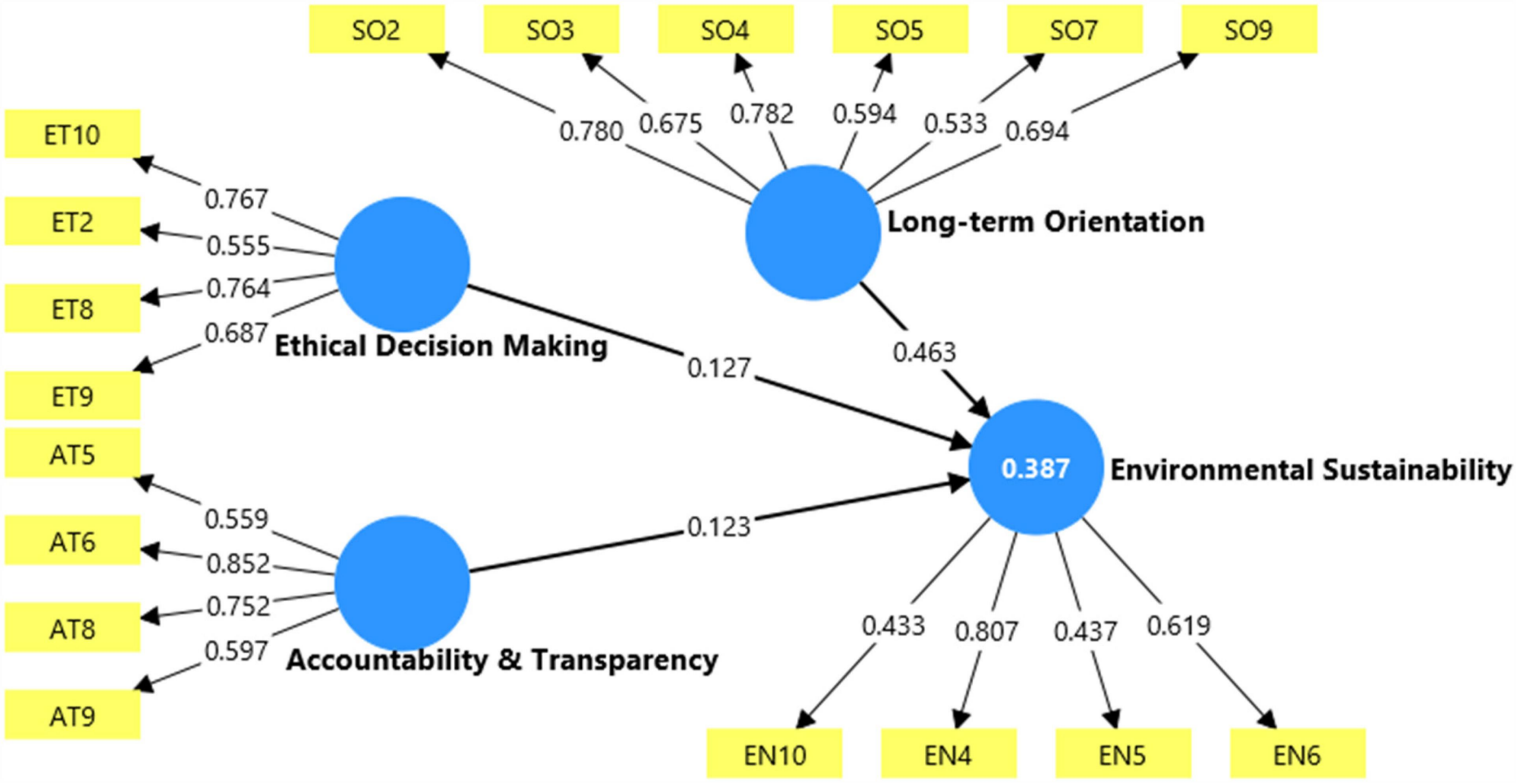
Environmental Sustainability model. Source: Author's own work.

The relationships between responsible leadership dimensions and environmental sustainability are shown in Figure 4.

Figure 4 illustrates the relationships between responsible leadership dimensions and environmental sustainability. The results indicate that responsible leadership explains 38.7% of the variance in environmental sustainability (R² = 0.387). This suggests that leadership practices play a substantial role in shaping environmentally responsible project outcomes. Among the predictors, long-term orientation again demonstrates the strongest predictive influence, reflecting the importance of forward-looking environmental planning and resource conservation. Ethical decision making and accountability and transparency also show positive contributions but with relatively lower predictive strength.

### Social sustainability model

**Figure 5:**
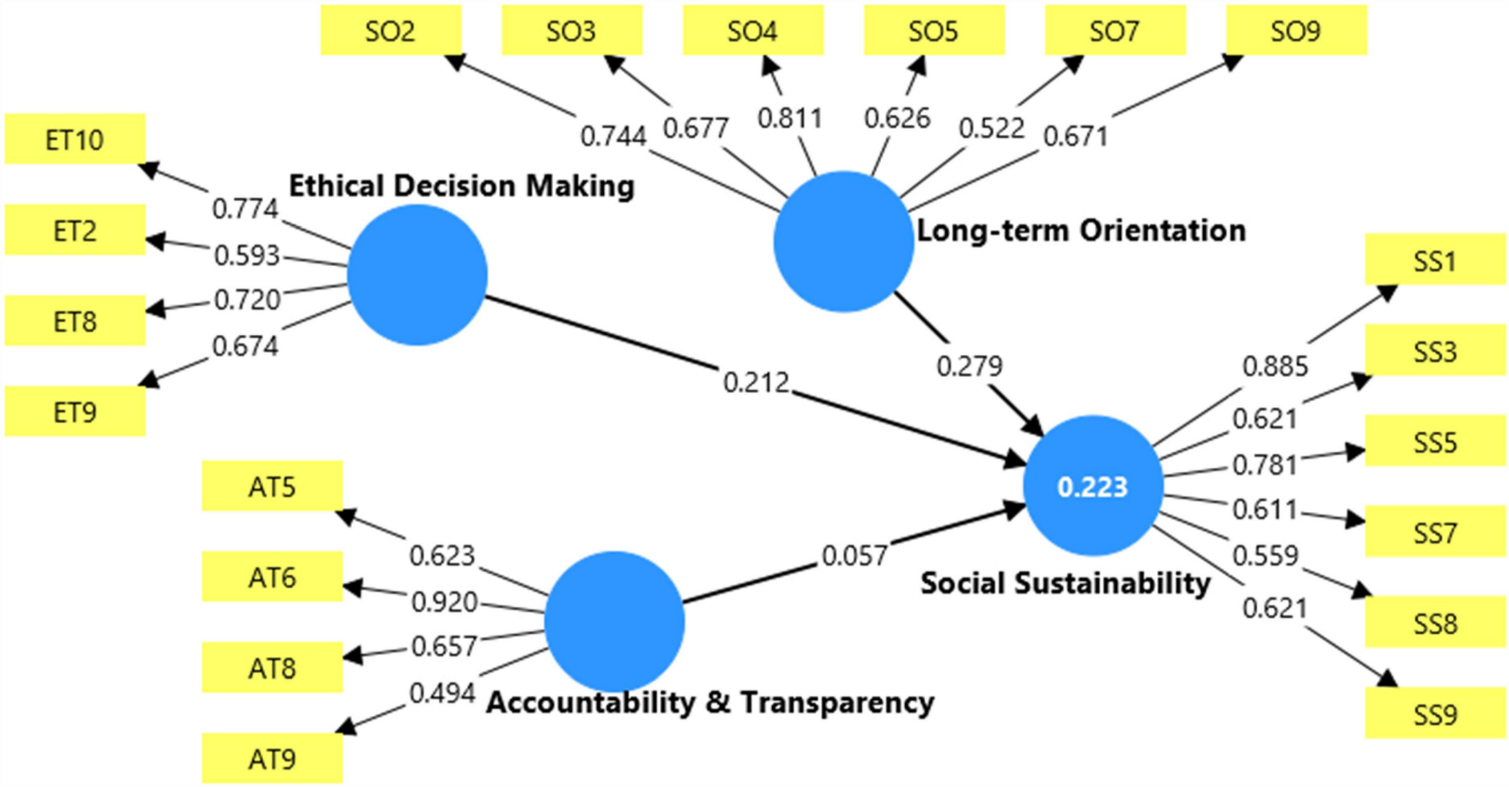
Social Sustainability model. Source: Author's own work.

The relationships between responsible leadership dimensions and social sustainability are presented in Figure 5.

Figure 5 presents the structural relationships between responsible leadership dimensions and social sustainability outcomes. The results show that responsible leadership explains 22.3% of the variance in social sustainability (R² = 0.223). Similar to the other sustainability dimensions, long-term orientation provides the strongest predictive effect, suggesting that leaders who prioritize long-term societal benefits enhance social sustainability outcomes. Ethical decision making also contributes positively to improving social outcomes such as community well-being and stakeholder inclusion, while accountability and transparency demonstrate a smaller but still positive influence.

**Figure 6:**
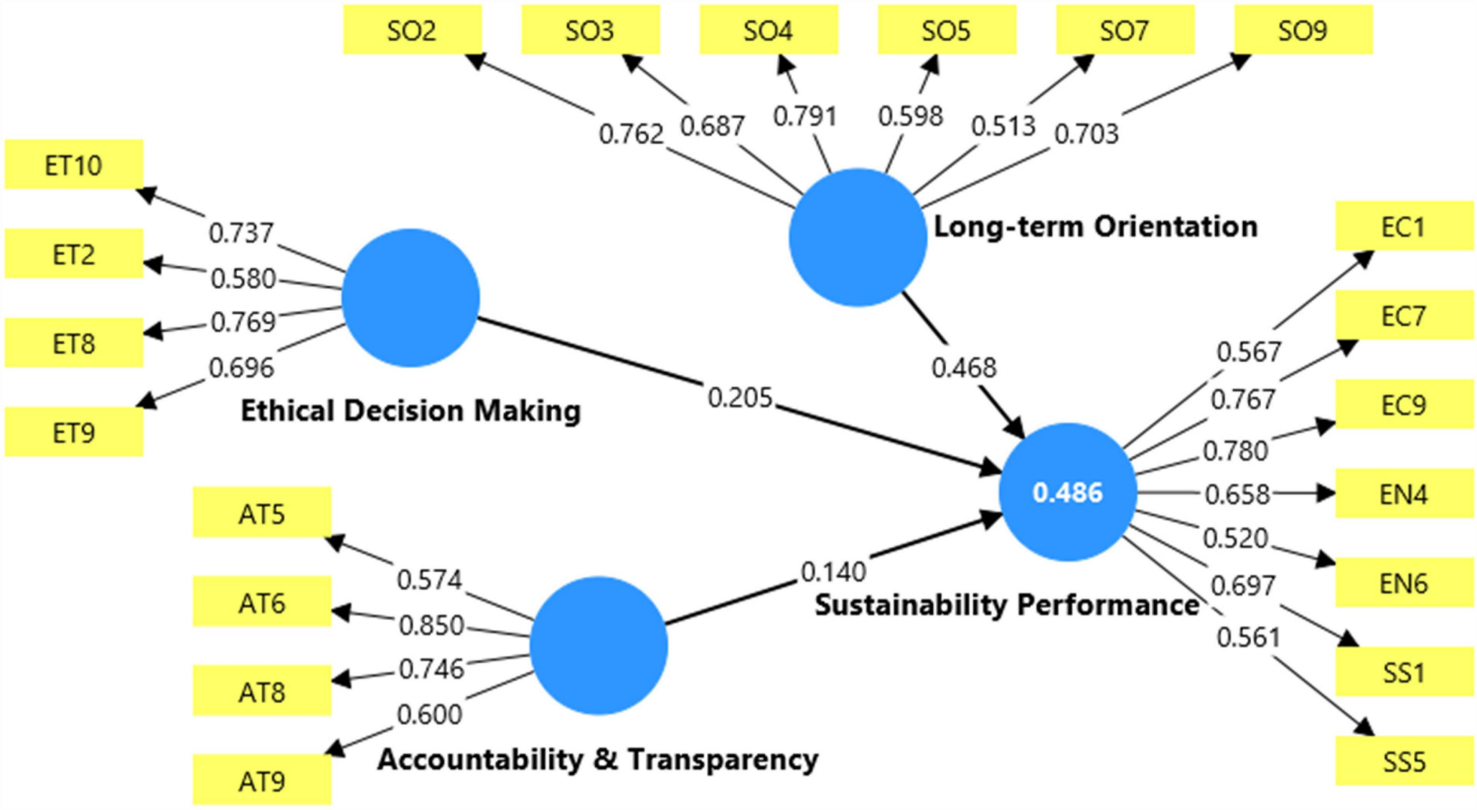
Responsible leadership practices and sustainability performance model. Source: Author's own work.

After confirming the adequacy of the measurement model, the structural model was evaluated to test the hypothesized relationships. The structural relationships between responsible leadership dimensions and sustainability performance are illustrated in Figure 6.

Figure 6 illustrates the overall structural relationship between responsible leadership practices and sustainability performance. The results indicate that responsible leadership practices when combined explains 48.6% of the variance in sustainability performance (R² = 0.486), demonstrating strong explanatory power. Among the responsible leadership dimensions, long-term orientation shows the strongest predictive effect on sustainability performance, followed by ethical decision making, while accountability and transparency exhibit a comparatively smaller influence. These findings suggest that leaders who adopt a long-term strategic perspective and make ethically grounded decisions are more effective in enhancing sustainability outcomes in health and sanitation projects.

PLS-Predict was used to evaluate the out-of-sample predictive power of the structural model, following the procedure recommended by Shmueli et al. (56). As presented in Table 9, the PLS prediction errors (RMSE) for all indicators are lower than those of the linear model (LM) benchmark, indicating that the model demonstrates superior predictive performance. Furthermore, all Q²_predict values are positive, ranging from 0.287 to 0.356, confirming that the model has strong predictive relevance for sustainability performance indicators.

**Table 9:** PLS-predict results (predictive power assessment).

Indicator	PLS Prediction Error (RMSE)	LM Benchmark RMSE	Q²_predict
EC1	0.492	0.521	0.331
EC7	0.468	0.506	0.364
EC9	0.455	0.498	0.371
EN4	0.501	0.534	0.309
EN6	0.523	0.556	0.284
SS1	0.489	0.516	0.336
SS5	0.511	0.540	0.298

Source: Author's own work.

EC, economic sustainability items; EN, environmental sustainability items; SS, social sustainability items.

### Hypothesis testing

The structural model was evaluated using the bootstrapping procedure with 5,000 resamples, as recommended by Hair et al. (45). The results presented in Table 10 shows that all hypothesized relationships are positive and statistically significant. Responsible leadership practices including ethical decision-making, accountability and transparency, and long-term orientation combined exhibits a strong positive effect with R-square predicting 48.6% of sustainability performance, providing support for H1. This indicates that leadership practices emphasizing ethical governance, transparency, and long-term orientation significantly enhance sustainability outcomes in health and sanitation projects. Ethical decision-making also demonstrates a significant positive effect (*β* = 0.205, t = 3.914, *p* < 0.001), supporting H2. This suggests that leaders who prioritize fairness, integrity, and responsible decision-making contribute positively to sustainability performance. Similarly, accountability and transparency show a positive and significant influence on sustainability performance (*β* = 0.140, t = 2.763, *p* < 0.01), confirming H3. This finding highlights the importance of transparent governance mechanisms and accountability practices in enhancing project sustainability. Finally, long-term orientation exerts a significant positive effect on sustainability performance (*β* = 0.468, t = 5.112, *p* < 0.001), supporting H4. This suggests that leaders who prioritize long-term planning and sustainability objectives are more likely to achieve sustainable project outcomes. The effect size statistics indicate that responsible leadership demonstrates a moderate effect on sustainability performance, while ethical decision-making, accountability, and long-term orientation show small to moderate effects.

**Table 10:** Structural model estimates.

Relationship	*β*	t-value	*p*-value	f²	Decision
ET → SP	0.205	3.914	0.000	0.084	Supported
AT → SP	0.140	2.763	0.006	0.043	Supported
SO → SP	0.468	5.112	0.000	0.121	Supported

Source: Author's own work.

ET, ethical decision-making; AT, accountability and transparency; SO, long-term orientation; SP, sustainability performance.

## Discussion

This study examined the predictive role of responsible leadership on the sustainability performance of health and sanitation projects in Uganda. All four hypotheses were supported, indicating that responsible leadership practices and its core dimensions such as ethical decision-making, accountability and transparency, and long-term orientation positively and significantly influence triple bottom line sustainability performance. These findings reinforce the argument that leadership behaviors are not peripheral but central drivers of sustainability in project-based public health interventions.

The positive and significant relationships between the dimensions of responsible leadership practices (including ethical decision-making, accountability and transparency, and long-term orientation combined) and sustainability performance support previous research demonstrating that responsible leadership practices grounded in stakeholder engagement and ethical responsibility enhance sustainable outcomes (12, 25). The findings are consistent with Ur Rehman et al. (11), who reported that responsible leadership improves financial and environmental performance, and Liu et al. (13), who found that responsible leadership promotes sustainable performance through pro-environmental behaviors. Similarly, Lin et al. (14) and Yu et al. (15) showed that responsible leadership practices strengthen collective and social responsibility performance in project-based contexts. Unlike most previous studies conducted in manufacturing, construction, or hospitality settings, this study demonstrates that the individual dimensions of responsible leadership significantly contribute to sustainability performance within health and sanitation projects in a developing-country context. Specifically, ethical decision-making enhances fairness and integrity in project implementation, accountability and transparency strengthen governance and stakeholder confidence, while long-term orientation promotes strategic planning and sustained value creation. Collectively, these findings suggest that responsible leadership practices enable project leaders to integrate ethical standards, transparent governance, and future-oriented planning into improved sustainability outcomes despite the complex stakeholder relationships, funding constraints, and accountability demands that characterize public health projects. Consequently, the findings provide empirical support for the relational and stakeholder-based perspectives of leadership advanced by Maak and Pless (8) and Waldman et al. (10), demonstrating that sustainability performance is strengthened through responsible leadership practices enacted via their distinct but complementary dimensions.

The significant positive effect of ethical decision-making on sustainability performance underscores the centrality of moral judgment and fairness in project sustainability. This finding is consistent with Khanam et al. (26), who found that ethical climates mediate the relationship between responsible leadership and sustainable performance, and Lu et al. (36), who demonstrated that responsible leadership promotes environmentally responsible behavior through ethical influence. Abraham (2) also emphasizes that ethical orientation is foundational to achieving triple bottom line outcomes. In health and sanitation projects, ethical decision-making may manifest through equitable resource allocation, prioritization of vulnerable communities, and compliance with environmental safeguards. The results suggest that when leaders make fair and transparent decisions, projects are more likely to achieve sustained economic efficiency, social acceptance, and environmental stewardship. This finding is particularly important in public-sector settings where ethical lapses can undermine community trust and long-term project viability. By empirically linking ethical decision-making to sustainability performance in Uganda's health and sanitation sector, this study addresses a contextual gap where leadership ethics had not been directly examined as a sustainability driver.

The findings also reveal that accountability and transparency significantly enhance sustainability performance. This supports Huo et al. (25), who highlight transparency as a mechanism through which responsible leadership strengthens sustainable outcomes, and Muff et al. (39), who identify accountability and stakeholder engagement as core competencies of responsible leaders. Transparent reporting and answerability reduce opportunistic behaviors and align stakeholder expectations, thereby improving economic and environmental outcomes (11). In the context of Uganda's health and sanitation projects where sustainability challenges have been linked to weak coordination and oversight (20), accountable leadership appears to mitigate governance gaps. Transparent communication regarding project finances, environmental safeguards, and service delivery strengthens stakeholder trust and promotes continued support. These findings move beyond structural governance mechanisms emphasized in prior Ugandan studies (16, 17) by demonstrating that leadership-driven accountability behaviors themselves directly influence sustainability performance. Although accountability and transparency significantly influenced sustainability performance, their comparatively smaller effect suggests that governance practices alone may be insufficient to guarantee sustainable project outcomes. While transparent reporting and accountability mechanisms improve stakeholder confidence and reduce opportunistic behavior, their effectiveness often depends on complementary leadership capabilities such as ethical decision-making and long-term strategic orientation. In the Ugandan public health context, accountability mechanisms are frequently constrained by institutional capacity, bureaucratic procedures, and resource limitations, which may reduce their direct influence on sustainability performance despite their importance for strengthening governance.

The positive influence of long-term orientation on sustainability performance reinforces the argument that sustainable outcomes require forward-looking leadership. (40) notes that sustainability-oriented leadership involves systems thinking and future-focused decision-making. Similarly, Liu et al. (13) and Ahsan (42) demonstrate that long-term strategic orientation enhances environmental innovation and sustained financial performance. In project settings, long-term orientation ensures that leaders integrate maintenance planning, environmental risk mitigation, and community capacity-building into project design (14, 43). The present findings suggest that when leaders prioritize future viability over short-term gains, health and sanitation projects are more likely to sustain financial stability, maintain infrastructure functionality, and preserve environmental resources. This is particularly relevant in Uganda, where short funding cycles and limited post-implementation planning have historically undermined project continuity (20). By empirically linking long-term orientation to sustainability performance, the study highlights the importance of strategic foresight in public health project management. The comparatively stronger effect of long-term orientation suggests that sustainability performance in health and sanitation projects is driven more by leaders' ability to prioritize future project continuity than by short-term operational considerations. Health and sanitation projects typically require continuous maintenance, institutional support, community ownership, and long-term resource planning to remain functional after external funding ends. Leaders who adopt a long-term perspective are therefore better positioned to integrate sustainability considerations into project planning, resource allocation, and stakeholder engagement, resulting in stronger economic, social, and environmental outcomes. This finding reflects the reality of Uganda's public health projects, where inadequate post-implementation planning has frequently contributed to project failure and infrastructure deterioration (20).

Collectively, the findings strengthen relational and responsible leadership theory by demonstrating that leadership behaviors enacted through ethical engagement, transparency, and long-term commitment translate into measurable sustainability outcomes in a project-based public health context (8, 9). The results extend prior firm-level studies (12, 25) into a neglected domain which is health and sanitation projects in Sub-Saharan Africa, thereby contributing context-specific evidence aligned with SDGs 3 and 6. Furthermore, while sustainability research in Uganda has largely focused on structural drivers such as governance systems, human capital, and management controls (3, 16, 17), this study demonstrates that leadership behaviors themselves serve as primary predictors of sustainability performance. The consistent positive effects across all four hypotheses suggest that responsible leadership is not merely supportive but foundational to achieving economic, social, and environmental sustainability in health and sanitation projects. Overall, the findings indicate that strengthening responsible leadership competencies among project managers and public health leaders may represent a strategic pathway for enhancing the long-term sustainability of health and sanitation initiatives in developing economies.

## Conclusion

This study examined the influence of responsible leadership on the sustainability performance of health and sanitation projects in Central Uganda. The findings demonstrate that responsible leadership operationalized through ethical decision-making, accountability and transparency, and long-term orientation significantly and positively predicts economic, social, and environmental sustainability outcomes. By situating leadership within a relational framework, the study confirms that sustainability performance in project-based public health interventions is not solely determined by structural governance systems or financial inputs, but is fundamentally shaped by leadership behaviors enacted through stakeholder relationships. The study therefore contributes both theoretically and empirically by extending responsible and relational leadership scholarship into the health and sanitation sector in a Sub-Saharan African context, offering evidence aligned with the broader objectives of sustainable development.

### Theoretical implications

This study advances Relational Leadership Theory (RLT) (29) by empirically demonstrating that leadership effectiveness in sustainability contexts is relationally constructed through ethical decision-making, accountability, transparency, and long-term orientation. RLT posits that leadership emerges from social interactions and stakeholder relationships rather than formal authority alone. The findings confirm that when leaders cultivate trust, fairness, openness, and future-oriented engagement, these relational processes translate into measurable triple bottom line outcomes. By extending relational leadership theory from corporate and manufacturing settings to project-based public health interventions in Sub-Saharan Africa, the study broadens the contextual boundaries of the theory. It shows that sustainability performance in health and sanitation projects is not merely a function of structural governance systems but is fundamentally shaped by relational leadership behaviors enacted across stakeholder networks. Thus, the study strengthens the predictive validity of RLT in explaining sustainability performance in complex, multi-actor development environments.

### Practical implications

Practically, the findings suggest that improving sustainability performance in health and sanitation projects requires deliberate investment in responsible leadership competencies. Government agencies, NGOs, and donor organizations should prioritize training programs that strengthen ethical decision-making, transparency practices, and long-term sustainability planning among project leaders. Embedding accountability mechanisms, open reporting systems, and stakeholder dialogue platforms within project governance structures can enhance trust and long-term viability. Furthermore, recruitment and performance appraisal systems for project managers should incorporate responsible leadership indicators alongside technical competencies. By institutionalizing relational and responsible leadership practices, policymakers and development partners can improve the economic efficiency, social inclusiveness, and environmental resilience of health and sanitation projects in Uganda and similar contexts.

### Limitations and future research

Despite its contributions, this study has several limitations. First, the cross-sectional design restricts causal inference, as data were collected at a single point in time. Future research may adopt longitudinal designs to examine how responsible leadership influences sustainability performance across different project life-cycle stages. Second, because responses were analyzed at the stakeholder level rather than aggregated to the project level, inter-rater agreement measures such as rwg and ICC were not assessed. Future studies may adopt multilevel designs and formal aggregation procedures to examine project-level effects more explicitly. Third, data were collected using self-reported measures, which may introduce common method bias despite procedural controls. Future studies could incorporate objective sustainability indicators, secondary project performance data, or multi-source assessments to enhance robustness. Third, the study focused on three districts in Central Uganda, which may limit generalizability to other regions with different institutional and socio-economic conditions. Comparative studies across regions or countries could provide broader insights. Finally, future research may explore mediating or moderating mechanisms such as stakeholder engagement, governance, or resilience, to further unpack how responsible leadership translates into sustainable outcomes in complex development settings.

## Data Availability

The original contributions presented in the study are included in the article/Supplementary Material, further inquiries can be directed to the corresponding author.
